# Traces of health—A landscape design task as a diagnostic aid for detecting mental burden? A qualitative focus group study

**DOI:** 10.3389/fpsyg.2023.1015169

**Published:** 2023-05-12

**Authors:** Christina Niedermann, Dennis Anheyer, Emily Seeligmüller, Thomas Ostermann

**Affiliations:** ^1^Department of Psychosomatic Medicine, Die Filderklinik, Filderstadt, Germany; ^2^Department of Psychology and Psychotherapy, Faculty of Health, Witten/Herdecke University, Witten, Germany; ^3^Department of General Practice and Interprofessional Care, University Hospital Tübingen, Tübingen, Germany

**Keywords:** mental health, prevention, art therapy, predictors, landscape design

## Abstract

**Background:**

Mental disorders are most common causes of illness worldwide. Studies on art and drawing tasks, such as the tree-drawing test have already proven their prognostic quality for the diagnosis of Alzheimer’s disease, depression or trauma. In the depiction of art in public space, gardens and landscapes are one of the oldest human forms of artistic expression. This study thus aims at exploring the impact of a landscape design task as a prognostic tool to detect mental burden.

**Materials and methods:**

A total of 15 individuals (eight female) aged between 19 and 60 years completed the Brief Symptom Inventory BSI-18 and the State Trait Anxiety Inventory (STAI-S) before being asked to design a landscape in a 3 × 3 m squared area. Material to be used included plants, flowers, branches, and stones. The complete process of landscape design was videotaped and the tapes were analyzed in a two-step focus group analysis from a group of gardening trainees, psychology students and students of arts therapies. Results were condensed in a second step into major categories.

**Results:**

Scores of the BSI-18 showed a range of 2–21 points and STAI-S scores ranged between 29 and 54 points indicating a light to moderate mental burden. Focus group participants identified three mutually perpendicular major components associated with mental health: “Movement and Activity,” “Material Selection and Design,” and “Connectedness to the task.” In a subsample of the three least and three most mentally stressed subjects (based on their GSI and STAI-S scorings), clear differences were found in body posture, action planning and the choice of material and aspects of design.

**Discussion:**

In addition to the well-known therapeutic potential of gardening, this study for the first time showed that gardening and landscape design contains diagnostic elements. Our preliminary findings are in coherence with similar research indicating a high association of movement and design patterns with mental burden. However, due to the pilot nature of the study, the results should be interpreted cautiously. Based on the findings further studies are currently planned.

## Background

Mental disorders are one of the most common illnesses in the population. Epidemiological studies suggest that the prevalence of mental illness among adults in Germany is around 30% (Nübling et al., 2014), with around 1–2% being classified as severely mentally ill (Gühne et al., 2015). In order to recognize corresponding signs at an early stage in the sense of prevention, health promotion and prevention represent important cornerstones in patient-oriented healthcare systems (Bauer et al., 2016).

In clinical psychology a variety of tests, classifications and procedures do exist to detect mental disorders, which are namely summarized in the Diagnostic and Statistical Manual of Mental Disorders (DSM-5) (Schneider et al., 2022). Most of them do include clinical tests (questionnaires) such as the PTSD Scale for DSM-5 (Marx et al., 2022) or the clinically useful depression outcome scale CUDOS (Li et al., 2021) which have been shown to be reliable and valid.

Apart from these test batteries clinical psychology also offers non-verbal approaches in which patients use art-based techniques like drawing, constellation work or construction tasks, which are commonly summarized under the term “projective tests.” These procedures have a long tradition in the diagnosis of mental burden in psychology. Already in the early days of psychological research, association studies were described by Galton (1879), Jung and Riklin (1906), Wundt (1907), which are considered the first precursors of projective test procedures.

While in classical projective methods such as Rorschach Tests the creative component of the participants is located in the verbal associations about the image, art based task consists of converting a simple task (i.e., drawing an object or a person) with use of appropriate materials into an artwork. Many of these procedures such as the House Tree-Person Test (Buck and Warren, 1970), the Draw A Person Test (Goodenough, 1926), the Sceno Test (von Staabs, 1995) emerged in the first half of the 20th century and are widely used in clinical contexts, especially when working with children or intellectual disabled persons (Lehmhaus and Reiffen-Züger, 2017; Neudecker and Horak, 2017). Due to their low-threshold, they are often used as an additional diagnostic aid (Schneider, 2018).

**FIGURE 1:**
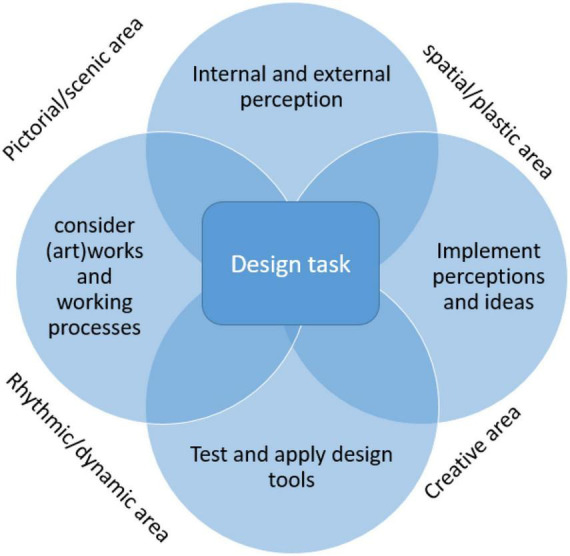
Coordinates of a design task adapted from Stöckli et al. (2016).

However, these tasks are more complex in terms of their evaluations. Here, creative aspects in relation to the implementation of ideas i.e., the use of specific materials, dynamic aspects of the work design (movement trajectories, activity, use of the available design space) play a central role. According to Stöckli et al. (2016) they can be attributed to the four dimensions “pictorial/scenic area,” “spatial/plastic area,” “Rhythmic/dynamic area,” and the “Creative area” (Figure 1).

Following this tradition, the design of a landscape might also represent an individual form of artistic expression, which first was noted this way by the well-known English poet and landscape architect Alexander Pope as “All gardening is painting” [Pope, quoted in Thelen (2014), p. 83]. Thus, if landscapes and their creation are understood as perceptual phenomena in the sense of realized images of idealized conceptions of landscape, landscapes could also be viewed as images (Petzold, 2011; Thelen, 2014) which, like images in art therapy, have an inherent diagnostic potential.

Similar to the drawing process itself, which in a digital tree drawing task has been shown to have predictive elements to detect mild cognitive impairment (Robens et al., 2019), the process of landscape design might also provide information about the mental burden of the designer as it is defined as a design process of outdoor spaces which “deals with form and meaning and is concerned with the organisation of a physical, functional and aesthetic arrangement of a variety of structural elements” (Nijhuis, 2013). Thus, identifying predictive elements in the landscape design process might lead to patterns which according to Pöppel (2015), p. 29, can be seen as “carriers of knowledge and cognition.”

The current qualitative study thus aims at identifying characteristic elements in an experimental landscape design task by art-therapeutic, psychological and horticultural experts using a focus group approach.

## Materials and methods

In a planting box with a size of 2.5 × 2.5 m, specially prepared for the study, work was carried out under video recording for a maximum of 30 min under the task “Design a landscape the way you want it!” The following materials were available:

• Building materials, such as sandstones, limestones, boulders, coarse gravel stones, various roots, branches and twigs of adjacent trees, reeds, willows.
• Miscellaneous plants such as four pcs. Verbenia, six pcs. Lavender, four pcs. Purple bellflower, four pcs. Houseleek, five pcs. Blue fescue, four pcs. Taxus media (Greenland), four pcs. Ivy, six pcs. tall candle spirea, various grasses.
• Various gardening tools such as shovel and rake, and work gloves.

**FIGURE 2:**
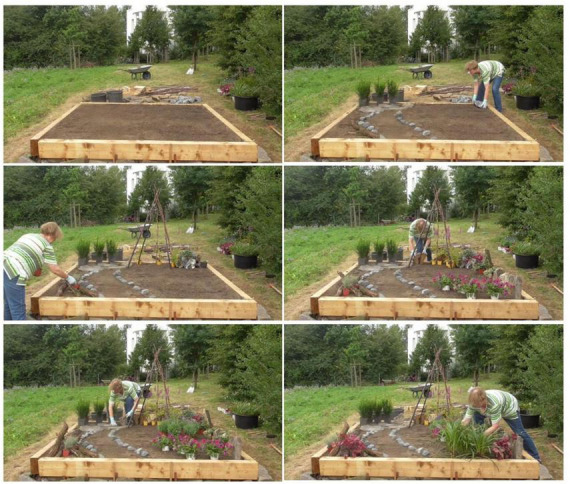
Experimental design and the process of the landscape design task.

Figure 2 gives insight into the experimental design and the process of the landscape design task.

### Ethical aspects

The study received a positive vote of the Ethics Committee for Art Therapies at Nürtingen-Geislingen University of Applied Sciences (HfWU) and was conducted according to the standards defined in the Declaration of Helsinki. Deletion of the data and withdrawal from the study was possible at any time at the request of the participants at any time without giving reasons and without incurring any disadvantages. Film recordings were conducted without giving any names and were transcribed anonymously and analyzed anonymously accordingly. The provisions of confidentiality and data security were guaranteed.

### Screening instruments for mental health

#### Brief symptom inventory 18 (BSI 18)

The BSI 18 is an 18-item self-report measure of psychopathological distress within the past 2 weeks (Franke et al., 2011; Spitzer et al., 2011). Individual differences are assessed with three six-item scales in the dimensions somatization (SOMA), depressiveness (DEPR), and anxiety (ANGS), as well as the global characteristic score GSI. A five-point Likert scale is used to record responses ranging from “0 = not at all” to “4 = very much.” For evaluation, summed values are calculated so that GSI ranges from 0 to 72 and for the three scales from 0 to 14. GSI values of 20 or more are considered clinically relevant. In a validation study, the psychometric values are Cronbach’s α = 0.88 for students, 0.82 for non-clinical subjects and 0.88–0.90 for clinical subjects. Test-retest reliability for the GSI is r = 0.77.

#### State trait anxiety inventory (STAI-S)

The STAI-S is based on the distinction between anxiety as a state (state-anxiety) and anxiety as a trait (trait-anxiety), i.e., an enduring characteristic (Laux et al., 1981). In contrast, anxiety as a momentary state refers to individual differences in stress with regard to anxiety reactions and is represented in the STAI-S with 20 items. On a four-point response scale from “1 = not at all” to “4 = very much,” a maximal total sum of 80 points can be achieved. In the original English version, cut-off values for a clinically relevant anxiety effect of 39–40 are suggested (Knight et al., 1983). Internal consistencies for both scales are α = 0.90; retest reliabilities for the state anxiety scale were between r = 0.22 and r = 0.53.

Together with the GSI, the STAI-S was used to determine the amount of mental burden of the participants. The ranking was primary based on the GSI-value. In the case of equal GSI values, the STAI-S value was used to arrive at an unambiguous order of the participants with respect to their mental burden. IBM SPSS version 26 was used to analyze the quantitative questionnaire data.

#### Sociodemographic and task related items

Participants were asked about their basic sociodemographic data i.e., age and gender. In addition, to assess task mastery, self-assessment in terms of gardening and artistic skills was asked on a visual analog scale from 0 to 10.

### Sample

Students as well as employees of the UW/H were recruited via a call for participation in this very study. Exclusion criterion was the presence of a strongly allergic or topical reaction to plant soil and/or plants. A total of 15 participants (eight female; Mean age: 28.20 years (SD = 12.78), Range: 19–60 years) took part in the study. The sample rated their gardening skills medium ($\bar{x}$ = 5.40; SD = 2.03) and their creativity high ($\bar{x}$ = 7.29, SD = 1.77). Psychopathological distress within the past 2 weeks measured with the GSI ranged from 2 to 21 points with a mean value of 7.93 (SD = 6.16) indicating a low to moderate mental burden. Value of the STAI-S ranged between 29 and 54 points with a mean of 37.6 (SD = 6.59), which is slightly below the clinically relevant cut off value of 40 points.

### Evaluation procedure

For the evaluation, we used a focus group approach. A focus group is a form of group discussion that is mainly used in qualitative social research. It is a moderated discussion of several participants based on the principles of communication and reflexion (Vogl, 2014; Bär et al., 2020). It is often used in early stages of study development to generate ideas, create concepts, and generate hypotheses. Focus groups have special goals and are therefore used to examine deeper attitudes, collective orientations–e.g., of the therapists or diagnostic statements.

Comparable to the study design of Heymann et al. (2018), a blinded-randomized analysis of the video material by a total of 56 experts from the fields “Gardening and landscape design” (GL) (*n* = 17, 3 female Mean age: 26.53 years); “Psychology and psychotherapy” (PP) (*n* = 15; 9 female; mean age: 23.07) and “Art therapy and Theater Pedagogy” (AT) (*n* = 24; 18 female; mean age 24.67 years) was conducted.

In a first step, the topic of the discussion was introduced to the experts by means of viewing a video of landscape design as a basis for discussion. The qualitative analysis was guided by the following questions: “What particular patterns do you see in the person’s landscaping activities?” and “What clues can you provide about whether these patterns are related to the person’s mental health?”. To avoid bias, experts were not introduced to the terms beforehand. The three groups of experts were interviewed separately on individual research days. Thus, there were three independent focus groups. The notes of the three focus groups were collected at the end of the day and evaluated in a second stage by another focus group.

**FIGURE 3:**
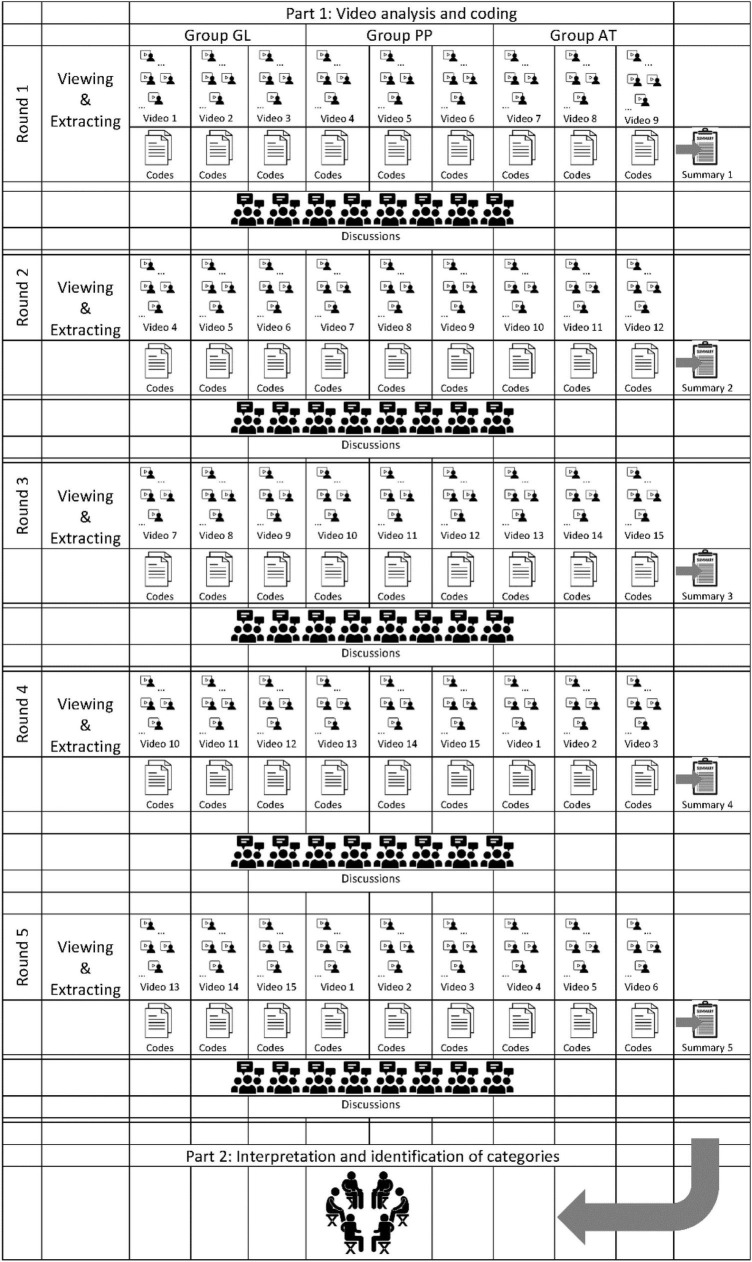
Summary of the single study steps and their interaction.

As shown in Figure 3, all 15 Videos were played to each expert individually, in full length and in original tempo as stimuli on a commercially available notebook. It was not possible for the participants to view other workstations or notebooks. During the video analysis, the experts were instructed to view the videos and to make notes on note sheets provided for this purpose. After 30 min in each sequence, the experts were asked to move to the next workstation.

Following three individual video viewings, an open discussion between freely chosen discussion partners took place in the context of a break situation. Notes were collected by CN and archived for documentation of the process and analyzing in Part 2 (see Figure 3). It was expected that a common mindset would emerge among the participants and that a synthesis effect first would occur (Croucher and Cronn-Mills, 2021). If a video was shorter than 30 min, the appropriate participants are asked to remain quiet at their respective workstations, complete notes, or take a break.

This first step was followed by a second more in-depth focus group to enhance the qualitative data generated, marked by “Part 2: Interpretation and identification of categories” in Figure 3 (Croucher and Cronn-Mills, 2021). Finally, an interpretation team consisting of five psychology graduates analyzed generated data. First, the team evaluated the qualitative data material of the experts in gardening/landscape design, in a purely inductive approach. A concept development was carried out on this first data set and respective notes were written. Then, iteratively a contrasting and increasingly deductive comparison was made using the second set of data from the experts in PP using the same task. This was done with the aim of establishing a theory generalized over the object of investigation in the third evaluation step on the data material of the experts in AT. Thus, the categories gain a higher validity. Each of the three evaluation rounds was followed by a joint focus group led by the first author (CN) in which all team members discuss the preliminary results and make hypothesis-generating interpretations with regard to a common understanding of the object of investigation.

For the evaluation of the qualitative data, a qualitative content analysis according to Glaser’s Grounded Theory (GT) (Strübing, 2014) was conducted. The dataset was iteratively read to develop first preliminary conceptual categories. For each of these categories, the patterns and characteristics were noted manually. In the course of this process, categories were merged, changed or removed to best fit the items. Finally, categories were grouped into broader, more abstract headings. Figure 3 summarizes the different steps of the study.

In a final evaluation, the categories found and their items were examined with regard to the mental burden of the subjects. For this purpose, the items for the three least and three most mentally stressed subjects (based on their GSI and STAI-S scorings) were analyzed again and differences were qualitatively worked out.

## Results

In total, the statements of 56 experts were screened for possible categories with regard to meaningful statements about mental burden. These statements were evaluated and grouped into the following three mutually perpendicular categories “Movement and Activity,” “Material Selection and Design,” and “Connectedness with the Task” were extracted as major categories. In addition, two general horizontal categories “Resonance” and “Overall impression in terms of fit/aesthetics” were added to this system by the interpretation team (Table 1). These two categories are thus exclusively the expression of a reflective evaluation process of the interpretation team.

**TABLE 1:** Major categories and their subcategories found by the focus group analysis.

Movement and activity	Choice of materials and design	Connectedness with the task
Body posture	Division of the area (in the sense of space grabbing)	Dialogue with the work
Movement sequence and endurance	Combination of materials	Reference to the work
Action planning	Color design	–
–	Representation	–
–	Material texture	–
**Resonance**		
**Overall impression in terms of fit/aesthetics**		

### Movement and activity

The major category “Movement and Activity” describes all visible movement patterns in the sense of a behavior and its underlying dynamics or vitality and includes three minor categories: “Body posture,” “Movement and Endurance,” and “Action Planning.” These categories link purely physical components such as the posture of the upper body or the shoulders with aspects of the movement of the represented physique and its dynamics.

#### Body posture

The category posture describes essential characteristics associated with psychological wellbeing with regard to the posture of the entire body, such as head position, shoulder position, muscle tone or leg position. In this context, the aspect of self-care was considered as an indication of mental health. This emerged primarily from healthy posture during work and can accordingly be represented dimensionally. For example, healthy posture is characterized by appropriate muscle tone, back-friendly working posture or sensible movement patterns:

“…*works in a back-friendly way, often squats down*…”

*(Code_Rater: MF28 (GL); Code_Participant: GD30)*

“…*very unhealthy posture when planting*…”

*(Code_Rater: BM16 (GL); Code_Participant: TN89)*

*“Stiff”*

*(Code_Rater: MK25 (AT); Code_Participant: BR26)*

Further, the interpretive team pointed out that entering the plant box and thus working inside the field might be associated with an emotional attachment to the task and inner balance. In contrast, working from outside the box would be associated with a higher emotional detachment from the task and one’s emotions, as well as a higher need for control and security, which could be associated with inner instability.

“…*Hardly enters the bedding area and works a lot from the back*

…” *(Code_Rater: MF28 (GL); Code_Participant: IU03)*

#### Movement sequence and endurance

Movement sequence and endurance described both the movement patterns and sequences initiated by the participant as well as the dynamics within. The body posture seems to have a significant influence on the sequence of movements:

“…*very determined, proceeds very neatly*…”

*(Code_Rater: BD24 (PP); Code_Participant: PP26)*

*“rather leisurely, slow working”*

*(Code_Rater: MF28 (GL); Code_Participant: CF01)*

“…*movements very uninspired*…”

*(Code_Rater: MF28 (GL); Code_Participant: TN89)*

*movements resembled “danccing” in parts*

*(Code_Rater: BM16 (GL); Code_Participant: CF01)*

From the statements, it became obvious that movement and motivation seem to be inextricably linked: Many statements about the course of movement or the speed of movement were always linked to an interpretation. Movement is apparently associated with a motivational situation between the poles of/inefficient/effortless/lustless/delayed/uncertain, via a median described as efficient/intentional/relaxed/satisfied/goal-oriented that was perceived as healthy, to a further pole of hectic /slow/unmotivated/less careful/associative/compulsive/impulsive. The movement process can increase to a visible flow experience due to this linkage.

#### Action planning

“Action planning” symbolizes the implementation of an idea, with particular emphasis on efficient vs. hesitant goal pursuit. A hesitant pursuit of goals is characterized on the one hand by a longer period until the start of the activity or the implementation of the planning and on the other hand by frequent interruptions of the (gardening) activity. Examples for codes relying to this category were:

*“sketches circles into the earth beforehand”*

*(Code_Rater: MK25 (AT); Code_Participant: BR26)*

*“Very indecisive/unstructured approach”*

*(Code_Rater: BM16 (GL); Code_Participant: TN89)Gives himself structures to follow*

*(Code_Rater: MK25 (AT); Code_Participant: BR26)*

Moments of interruption or pause were also identified and related to this category. The interpretation team understood such moments mainly more as a moment of indecision. It appears that the original planning was interrupted at this point and the pausing represents an adjustment of action. In some cases, further manipulation of already designed elements occurs after such moments. At the same time, a certain reflexive moment was seen, as illustrated in the following quote:

“…*takes a step back every now and then and walks around the area to get an overview”*

*(Code_Rater:BM16 (GL); Code_Participant: CF01)*

### Material selection and design

The major category “Material selection and design” consists of five minor categories: “*Structuring of the area,” “Combination of materials,” “Color design,” “Figurativeness,” and “Material texture.”* In summary it was found, that the meaningful and creative combination of diverse materials in terms of space use or color design is an indication of mental health. This meaningful and creative combination is associated with the concepts of harmony, fantasy or aesthetics. Unfortunately, no deeper explanation was given as to what was meant by the terms “creativity” or “aesthetics.”

#### Structuring of the area

The arrangement of the area refers to the spatial division and use of the area or the planting box, in the sense of taking up space. Here, the representation of symmetry has been given special importance. The interpretation team associated a strong symmetry with the need for control, insecurity and perfectionism. This is contrasted with asymmetry. Again, a kind of continuum emerges here, the center of which represents a dynamic composition of symmetrical and asymmetrical outline elements and is associated with mental health or inner balance and equilibrium. The middle of the box is often depicted here as the center representing the self of the person. Likewise, “empty spaces” are referred to, i.e., unworked or undesigned areas in the planter box that could be space for (visual) interpretation or places of rest:

*“Symmetries/mirroring of the planting across the “optical dividing line” in the center”*

*(Code_Rater: BM16 (GL); Code_Participant: SB07)*

*“Sticks and stones that break up the symmetry, loosen up, bring in liveliness”*

*(Code_Rater: HM26 (PP); Code_Participant: AA07)*

*“Arrangement of the surface seems to be done according to the golden ratio”*

*(Code_Rater: BM16 (GL); Code_Participant: EM10)*

*“Also separates the bed by color”*

*(Code_Rater: MF28 (GL); Code_Participant: PP26)*

#### Combination of material

This category captures the creative combination of different materials. Monotony is a description that is contrasted with diversity. Above all, the interpretation team referred to the meaningful combination of materials, which can, for example, result in concreteness. The quantity of materials is also placed by the interpretation team in a kind of bipolar relationship from “excessive amount of materials” to “meager choice of materials.” In addition to meaningfulness, the fit of the materials and their quantity is a decisive criterion that represents harmony in the sense of signs of mental health. Example codes were:

*“Designing the center with flowers and shrubs”*

*(Code_Rater: BK08 (PP); Code_Participant: AA07)*

*“Wood is used as a climbing aid for ivy”*

*(Code_Rater: BM16 (GL); Code_Participant: EM06)*

*“Stones are arranged around plants in a circle”*

*(Code_Rater: SS14 (PP); Code_Participant: AA07)*

#### Color design

Within this category the color design caused by the material selection is recorded. The interpretation team here again proposes a continuum with two poles consisting of “colorless” and “colorful”:

*“very colorful overall picture good mood”(Code_Rater: CI16 (PP); Code_Participant: PP26)*

*“Heuchera and ivy make good contrast by leaf color”*

*(Code_Rater: BM16 (GL); Code_Participant: AA07)*

*“creates a crazy colorful flowerbed”*

*(Code_Rater: HS24 (PP); Code_Participant: AA07)*

*“next to the circle single green color spots are placed”*

*(Code_Rater: RK03 (PP); Code_Participant: AA07)*

*“Bed very green and pink* = *green* + *pink radiant colors”*

*(Code_Rater: PF20 (AT); Code_Participant: BR26)*

#### Representationalism

Representationalism refers to the level of abstraction of the resulting garden painting. On the one hand, mandala-shaped portraits can arise, on the other hand three-dimensional landscapes, in which everyday objects were built by the use of materials and tools.

*“many geometric patterns. Squares surrounded by triangles surrounded by squares*…”

*(Code_Rater: FJ24 (PP); Code_Participant: AA07)*

“*Stone circle is planted with ivy; the garden looks more “alive””*

*(Code_Rater: GR22 (PP); Code_Participant: PP26)*

*“Wooden beam as stylized fence post forms exciting aspect”*

*(Code_Rater: BM16 (GL); Code_Participant: MB12)*

*“She designs a “wooden tippi” that she does not enter herself”(Code_Rater: GS22 (AT); Code_Participant: EK10)*

#### Material texture

Material texture describes the selection and type of material in terms of its properties and variety. Materials are described according to their physical properties as inanimate/dead (e.g., stone) or alive (e.g., plants). The integration of both materials is again seen by the interpretive team as an indicator of mental health. At the same time, inanimate material was associated with protection, safety, heaviness, barrenness, desolation, or stability. As a note, it must be pointed out here that it depends on how material is used and designed: depending on the material, lifeless material can have a protective but also a desolate effect. This again illustrates the diversity from the presentation of the focus group survey in the first step:

*“diverse overall picture with all kinds of different plants u materials contributing to a colorful appearance”*

*(Code_Rater: GR22 (PP); Code_Participant: PP26)*

*“Lots of flat plants, rocks, little height”*

*(Code_Rater: GR22 (PP); Code_Participant: PP26)*

*“extremely sparing use of material and plants”*

*(Code_Rater: BM16 (GL); Code_Participant: HJ21)*

*Sticks on the sides seem like foreign bodies, as do the sawed tree slides, too many different materials extremely restless/unstructured impression*

*(Code_Rater: BM16 (GL); Code_Participant: AA07)*

### Connectedness with the task

The major category “Connectedness with the task” consists of two categories, namely work dialogue and work reference.

#### Work dialogue

Work dialogue is intended to express the (ideational) exchange between the work and its author. It describes the (non-verbal) exchange between the gardening person and the landscape design. This is expressed, for example, by looking at the work, manipulating what has already been designed, or reconstructing design elements that have been thrown over.

*“proceeds very carefully and always makes sure that the plants relate to each other by their arrangement”*

*(Code_Rater: BD24 (PP); Code_Participant: PP26)*

*“seems to have plan in mind, again and again he looks at the whole field and balances”*

*(Code_Rater: BD24 (PP); Code_Participant: PP26)*

*“Dissolution of symmetry toward the end abstraction, own will/thinking”*

*(Code_Rater: TT11 (PP); Code_Participant: AA07)*

#### Work relatedness

Work relatedness, in turn, represents the state of identification of the person with the work or parts of it, understood in the psychoanalytic sense. It describes the personal involvement and of the gardening person with his or her work of landscape design.

*“shapes broadly no fears, open and not afraid of new things”*

*(Code_Rater: CM19 (PP); Code_Participant: PP26)*

*“flexibility and creativity in an orderly field indicate a good sense of wellbeing and self-confidence”*

*(Code_Rater: BD24 (PP); Code_Participant: PP26)*

*“creative mind with lack of concentration; chaos in the garden”(Code_Rater: HS24 (PP); Code_Participant: PP26)*

“*Design seems colorful Desire for versatility/vibrancy not harnessed in its design”*

*(Code_Rater: CM19 (PP); Code_Participant: PP26)*

### Resonance

Resonance of the rater describes the own reaction in the form of feelings, thoughts, impulses to the landscaping person himself or his garden artwork in the sense of a countertransference phenomenon.

*“Design makes the impression that the person has experience with gardening and can act out creatively and with pleasure”*

*(Code_Rater: GR22 (PP); Code_Participant: AA07)*

*“does not think long (little standstill, little pause, little looking at what has been created) appears lively easily”*

*(Code_Rater: HM26 (PP); Code_Participant: PP26)*

*“after he has created the order and structure, he can proceed freely, he seems to enjoy the creative possibilities and he appears confident and determined”*

*(Code_Rater: BD24 (PP); Code_Participant: PP26)*

*“inner order or compulsive adherence to a system”*

*(Code_Rater: DE09 (AT); Code_Participant: BR26)*

*“Drawing of a glove and plants, framed, in front of it “We protect this” with arrows pointing to the plants”*

*(Code_Rater: KI55 (AT); Code_Participant: BR26)*

Furthermore, with regard to the category “Overall impression in terms of fit/aesthetics,” it was found that both the overall impression in terms of the entire process as well as the resulting landscape image was seen in a spectrum between aesthetics and anti-aesthetics, respectively between beauty and ugliness.

*“wild but not unpleasant picture, ordered chaos”*

*(Code_Rater: GR22 (PP); Code_Participant: PP26)*

*“Planting also well-planned and executed, very harmonious not wildly jumbled together”*

*(Code_Rater: MF28 (GL); Code_Participant: EM10)*

*“very lively, partly restless and contradictory design”*

*(Code_Rater: TT11 (PP); Code_Participant: AA07)*

It has to be noted that the term “aesthetics” was used here in a very specific way and is equated with the colloquial usage “beautiful work.”

### Connection of the categories to mental health

In a final step, the items of the three participants with the highest mental burden were compared with those of the three participants with the lowest mental burden and analysed based on the categories given above. Table 2 summarized the main findings of this analysis.

**TABLE 2:** Characteristics of participants with low/high mental burden subdivided by the categories derived from the interpretation team.

	Low mental burden	High mental burden
Movement and activity		
Body posture	➢ “working out of the back” ➢ “flexed”	➢ “working out of the legs” ➢ “upright attitude”
Movement sequence and endurance	Movement sequence ➢ “hesitant” ➢ “relaxed” Working speed ➢ “slow”	Movement sequence ➢ “hesitant” ➢ “strained” Working speed ➢ “slow”
Action planning	➢ “goal oriented” ➢ “careful”	➢ “aimless” ➢ “uncoordinated”
Choice of materials and design	➢ “structured, coherent approach”	➢ “symmetrical”
Division of the area (in the sense of space grabbing)	➢ “Focus on peripheral areas” ➢ “creates islands”	➢ “starting in the middle” ➢ “Circular arrangement”
Combination of materials	➢ “Mainly plants and plant material (sticks)”	➢ “Mainly stones and flagstones”
Color design	No uniform image: from ➢ “colorful” to ➢ “monotonous”	No uniform image: from ➢ “colorful” to ➢ “monotonous”
Representation	Has not been coded sufficiently	Has not been coded sufficiently
Material texture	➢ “lively”	➢ “inanimate”
Connectedness with the task	➢ “Looking at the big picture” ➢ “focused”	➢ “Careful handling of the material” ➢ “focused”
Resonance	Has not been coded sufficiently	Has not been coded sufficiently
Overall impression	No uniform picture: from ➢ “harmonious” to ➢ “little aesthetics”	Has not been coded sufficiently

As was already evident in the unstratified analysis, there was a clearly discernible range in the coding with regard to some categories. In this second analysis, differences could partly be associated with a high or low mental burden. For example, in the category “Body Posture” raters of often described the working position of the participants with low mental burden as “flexed” while high mental burden was associated with a more “upright” and possibly even distanced position. With respect to “Action planning” participants with low mental burden were described as “goal oriented” and “careful” while participants with high mental burden were described as “aimless” and “uncoordinated.” Looking at the “Choice of materials and design” participants with low mental burden focus on peripheral areas and started creating “islands” mainly using plants and plant material (sticks), giving the landscape a more lively look in contrast to those with high mental burden who started working in the middle with “circular arrangements” mainly using stones and flagstones resulting in a more inanimate picture. According to the ratings, low mentally burdened participants connected to the task from a more global perspective “Looking at the big picture,” while on the other side a “careful handling of the material” was observed. No differences however, were found in workings speed, color design or focus. Finally some categories, e.g., “Resonance with the work” or “Overall impression” were not coded sufficiently in the selected participants.

## Discussion

Garden therapy represents a well-established therapeutic intervention for which there is extensive evidence of effectiveness presented in systematic reviews and meta-analyses (Clatworthy et al., 2013; Spring, 2016; Soga et al., 2017). In addition to this well-known and established therapeutic potential, this study for the first time showed that gardening and landscape design contains diagnostic elements.

The evaluation in two-stage focus groups produced three top categories, Movement and Activity, Material Selection and Design, and Connectedness to the Task, which are significantly interpretive in nature for capturing the psychological state of a landscaping individual. These categories can also be found in the literature (Lazar et al., 2018; Piccininni et al., 2018; Rohani et al., 2018; Choe et al., 2020).

In terms of material selection and design, some parallels could be found with evaluation results of a digital drawing task in which individuals with Mild Cognitive Impairment (MCI) and incipient Alzheimer’s dementia could be distinguished from a healthy comparison sample by components such as less color selection, less stroke width, less color and stroke width change, and less contrast, image size, and complexity (Robens et al., 2019). Also, the results provide evidence of abnormalities in the process analysis: individuals with MCI or AD onset persisted for longer periods of non-drawing, in the sense of pausing or procrastinating, and were significantly slower in the drawing process compared to the healthy comparison sample. In the present study, participants with high mental burden also were described as aimless in their goal planning and showed an uncoordinated behavior with stained movement sequences. Although not a complete match, the patterns are comparable to some degree. Quite surprisingly and not in line with previous findings, colorfulness of the landscape in our final evaluation was not associated with either low or high mental burden. However, it must be taken into account that the Drawing Task Study investigated Alzheimer’s patients, whereas the present study involved healthy subjects.

In terms of movement and activity it can be noted that, the speed of head movement is lowered in depressed patients (Girard et al., 2014). Studies in posture show that an upright sitting posture, as opposed to a slumped sitting posture, is associated with higher self-esteem, increased attention, elevated mood, as well as less anxiety (Kacem et al., 2018). In another analysis, Alghowinem et al. (2013) found the following characteristics in depressed patients: (a) slower head movement, (b) less change in head position, (c) looking to the right side for longer periods of time, and (d) looking down for longer periods of time. The authors interpreted the latter two items as an indicator of fatigue and avoidance of eye contact. Gait patterns associated with sadness and depression are more often associated with slower walking speed, less arm swinging, and vertical head movement (Michalak et al., 2009). Depressed and sad individuals sway more laterally in the upper body and show slumped posture in gait (Michalak et al., 2014). Again, our finding only partly go alongside with these results. While action planning pointed in a similar direction, movement speed did not differ in our ratings.

Also with respect to the “Connectedness with the task,” literature shows that the positive correlation between time spent in an allotment garden and eudemonic wellbeing is mediated entirely by the feeling of connectedness with nature (Webber et al., 2015). Here we were able to find differences: participants with lower mental burden connected to the task as a whole, while participants with high mental burden mainly focused on small units and details. This aspect is closely connected to the category “Resonance” and probably has to be sharpened in future investigations. Nevertheless, when talking about resonance, it can be assumed from the codings that the concept of resonance coined by Rosa is meant here, which is “about a precarious response event that the creator experiences as an artistic struggle” (Buschkühle, 2017), which ultimately leads to being “touched” by the work. Thus, Resonance is not only the connection with the task but refers to a relationship of (inner) dialogue with the work.

From a methodological perspective, the present approach is based on the procedure of Ruddat (2012) and contributes to generating further hypotheses for both the clinical-psychological diagnostics of garden and landscape design and the evaluation of art therapies. With the methodology used here, a first approach for further studies is given. One possible direction of research can be found in the field of dementia diagnostics. Here, an observation tool based on a tablet has been developed that allows to capture momentary changes in engagement during an activity by means of the Video Analysis Scale of Engagement (VASE) (Daniel Lai et al., 2020).

The use of focus groups in qualitative research has increased, especially in social science but also in health care with the aim of generating knowledge about yet unknown topics (O’Brien and Varley, 2012; Bär et al., 2020). In the literature, a unified notion of qualitative research methodology continuously seems to be established, which is characterized by methodological diversity and flexibility regarding its application (Kim et al., 2017).

In the field of arts therapies, focus groups, including MMR designs, are commonly used instruments to capture specific (effect) factors of arts therapies. For example, focus groups have been applied to analyze general effects in specific learning and experiential processes of art therapists in the handling and therapy of people with psychotic disorders, thus making them available for art therapy education (Holttum et al., 2021).

Other studies have used focus groups in terms of a reflexive tool. For example, the quantitative results of a study on the effect of art therapy on trauma-associated symptomatology in refugees were presented to clinicians and therapists and discussed in focus groups (Rowe et al., 2017). This study found that the individual, but also relevant general aspects of therapy success and underlying mechanisms could not be brought to light with the purely quantitative evaluation due to the high specificity and the underlying deductive research approach.

From a broader methodological point of view, qualitative approaches such as focus groups or Delphi processes can also be used to assess the development of artworks of clients (Schweizer et al., 2019). In particular, by observing the process of creating an artwork the focus group approach can also be used in the evaluation of the therapeutic process of a single client over the course of time. Furthermore as realized in an evaluation study of a peer support model of community wellbeing for mental health participants might also be included in a focus group approach to directly share their experience (Hardy et al., 2019). This approach was also realized in the exploration of effects of a dance/movement program on mental health for female survivors of intimate partner violence (Özümerzifon et al., 2022). Here, in contrast to our approach, the aim of the focus group was to collectively discuss the experiences during the artwork process and thus might serve as an additional source to capture the inner dimensions of the process.

## Limitations

Limitations of this study are firstly given by its generalizability, which is limited primarily by the small number of participants, although a certain heterogeneity can be observed in the sample with regard to age and professional experience. However, no valid recommendations for the use in the clinical-psychological setting can be made at this stage. Furthermore, with regard to the first part of the focus group survey, the inclusion criterion of experts is a matter of discussion. It can be assumed that more meaningful results would have been brought to light by persons with a more valid expert status, e.g., exclusively professionals with many years of practical experience. At the same time, it should be noted that the experts would have benefited from being interviewed at the same time, e.g., as part of a World Café method (Jong et al., 2019). However, and in line with findings of Vasileiou et al. (2018), it can critically be assumed, that In a theoretical saturation might not have been reached, i.e., additional insights could have been emerged from the data in particular when comparing the participants with high and low mental burden.

Methodologically, it can be critically noted that qualitative approaches such as the grounded theory used here might result in unreproducible results and interpretations. However, methodological comparisons e.g., with topic modeling show a “surprising degree of alignment in the findings” although the respective processes are different (Baumer et al., 2017). Thus we believe that our methodological approach might nevertheless results in compelling interpretations.

Regarding the analysed material, in the present study video material was chosen as the stimulus material for the focus groups. This of course has had a tiring effect on the participants. At the same time, the video is complex as a multimodal data carrier and may require a more detailed evaluation strategy. From the point of view of content, the limitation is given by to the non-clinical sample. The proposed category system thus only serves as a basis for further research.

## Future directions

Future studies even more than the study presented here should combine qualitative and quantitative elements of evaluation in order to arrive at a more comprehensive assessment of such approaches as described in Goodall et al. (2019). They should also use the framework of the design task as described by Stöckli et al. (2016) (Figure 1) even more precisely for theory building in order to generate further mixed methods study ideas from it. For this purpose, the digital landscape assessment tool developed by Roth (2012) can be used for a dynamic analysis of the garden design task carried out here. Even though this is currently only intended for larger areas than the planter boxes described here, it could also be promisingly used for the design task used here after appropriate modification. In particular, this index can also be used as a change marker for psychological wellbeing or changes in quality of life by assessing a total of 25 assessment criteria, such as character, diversity, beauty, closeness to nature, can also be used as change markers for psychological wellbeing or changes in quality of life (Roth and Gruehn, 2010).

With respect to human movement analysis, the use of Laban Movement Analysis (LMA) is another technique, which could be used for describing and evaluating human motion in a more systematic way, as already described in Durupinar et al. (2016) for the analysis of human movement and personality. This however, would require to recruit a group of certified Movement Therapist trained in the usage of LMA. Another innovative and not so personnel-intensive approach would be to have the LMA performed by artificial intelligence (AI). A recently conducted study on AI-driven Movement Analysis Using LMA found that such a system was able to annotate human motion in the dimensions Effort, Space, Shape and Body (Guo et al., 2022), which partly corresponds to the dimensions found in this study and therefore gives rise to promising results when applied in a similar context.

Finally it would be possible to install corresponding planting boxes in various facilities, e.g., elderly care, and to document the progress in gardening of residents of these facilities continuously (e.g., on a daily basis) in an automated manner. Appropriate nature-based offerings are already being used in multidisciplinary contexts in the field of elderly care (Gagliardi and Piccinini, 2019).

Additional quantitative analyses of the present video material with respect to movement entropy found a distinct gender effect, with a correlation of lower movement entropy in a landscaping task with a higher mental burden for men, but a lower mental burden for women (Unger et al., 2021a,b). Thus, the question of gender which has recently been raised with respect to the role of the natural environment in supporting population health and wellbeing in Colley et al. (2022) should also be focused in future research.

## Conclusion

According to a recent systematic literature review (Masterton et al., 2020), green space interventions provide promising opportunities for person-centered public health interventions. Specifically designed programs made available at the community level to a large group of people from different cultural as well as social backgrounds can therefore contribute not only to an increase in the quality of life and integration of disadvantaged parts of the population (Hou, 2017) but also to a generation of a potential tool for clinical diagnosis.

## Data availability statement

The original contributions presented in this study are included in the article/supplementary material, further inquiries can be directed to the corresponding author.

## Ethics statement

The studies involving human participants were reviewed and approved by Ethics Committee for Art Therapies at Nürtingen-Geislingen University of Applied Sciences (HfWU). The patients/participants provided their written informed consent to participate in this study.

## Author contributions

CN was responsible for the conduction of the study, analysis and investigation, wrote the first draft of the manuscript, and guided the focus group raters. DA and ES were responsible for the extraction and interpretation of characteristics of participants with low/high mental burden subdivided by the categories derived from the interpretation team. TO was responsible for the methodology of the study and prepared the final manuscript. All authors contributed to the article and approved the submitted version.
